# The Effect of Multidimensional Motivation Interventions on Cognitive and Behavioral Components of Motivation: Testing Martin's Model

**Published:** 2017-04

**Authors:** Fatemeh Pooragha Roodbarde, Siavash Talepasand, Issac Rahimian Boogar

**Affiliations:** 1 Faculty of Education and Psychology, Semnan University, Semnan. Iran.; 2 Department of Clinical Psychology of Semnan University, Semnan, Iran.

**Keywords:** *Behavioral*, *Cognitive*, *Motivation*, *Martin's Model*

## Abstract

**Objective:** The present study aimed at examining the effect of multidimensional motivation interventions based on Martin's model on cognitive and behavioral components of motivation.

**Method:** The research design was prospective with pretest, posttest, and follow-up, and 2 experimental groups. In this study, 90 students (45 participants in the experimental group and 45 in the control group) constituted the sample of the study, and they were selected by available sampling method. Motivation interventions were implemented for fifteen 60-minute sessions 3 times a week, which lasted for about 2 months. Data were analyzed using repeated measures multivariate variance analysis test.

**Results:** The findings revealed that multidimensional motivation interventions resulted in a significant increase in the scores of cognitive components such as self-efficacy, mastery goal, test anxiety, and feeling of lack of control, and behavioral components such as task management. The results of one-month follow-up indicated the stability of the created changes in test anxiety and cognitive strategies; however, no significant difference was found between the 2 groups at the follow-up in self-efficacy, mastery goals, source of control, and motivation.

**Conclusion:** The research evidence indicated that academic motivation is a multidimensional component and is affected by cognitive and behavioral factors; therefore, researchers, teachers, and other authorities should attend to these factors to increase academic motivation.

With respect to education, motivation is a multidimensional structure associated with learning and academic achievement and includes individual's beliefs about his/her ability to do a desired activity, reasons or goals, and emotional reaction related to that activity(1). The findings of the present study on academic motivation are derived from number of approaches including self-efficacy, expectancy, value theory, attribution theory, goal orientation, self-regulation, and the need to progress theory(2). Multidimensional approaches are one of the existing approaches for identifying the factors that affect motivation. Pintrich has proposed a multidimensional approach in studying motivation that includes self-efficacy, attributions, valuing, control, self-regulation, being purposeful, the need for progress, and self-valuing(3).

In addition, Martin proposes a cycle called cycle of motivation and academic involvement in academic motivation that encourages students to be involved in academic issues; this cycle has 4 behavioral and cognitive compatible and incompatible parts (4). He used several cognitive and behavioral theories of motivation to identify the components of this cycle and each of its levels.

they are as follow: self-efficacy theory: it includes the component of self-efficacy and attribution theory; control: it includes the component of attribution and locus of control; valuing theory: it reflects valuing; self-determination theory: it reflects the dimension of goal or motivation orientation; the need to progress and self-worth theory: it includes components such as failure avoidance, anxiety, self-handicap and not involving in academic activities;

self-regulation theory: it includes components such as planning, task management, and resistance (4). He used various cognitive and behavioral theories of motivation to identify the components of this cycle and each of its levels. In Martin's theory, these components are divided into 4 dimensions including compatible cognitive dimension (value, orientation, and self-efficacy), incompatible cognitive dimension (anxiety, avoidance of the failure, and the feeling of lacking control), compatible behavioral dimension (persistence, planning, and task management), and incompatible behavioral dimension (not involving in academic activities and self-effecting). In fact, these researchers attempted to develop a multidimensional and a more comprehensive point of view on motivation. Accordingly, in the present study, based on Martin and Pintrich's multidimensional motivations model and the results of the primary researches in this regard ( 5,6,7), 5 factors affecting students' academic motivation such as self-efficacy, mastery goal, test anxiety, feeling of lack of control, and task management were selected for practical examination. Self-efficacy is one of the important components in Martin's theory and Bandura's social-cognitive theory (8). Bandura believes that self-efficacy plays an important role in predicting individuals' success in various fields such as school, work, and relations (8, 9). Moreover, individuals who have high self-efficacy use a deeper process and reasoning and show more cognitive involvement in doing tasks (10). The theoretical bases were experimented in various fields and environments (11). Goal orientation also appeared as one of the important point of views in achievement motivation, especially academic motivation (12). Goal orientation as a criterion refers to the evaluation of learners' ability and their progress towards the goal and the reasons that involve the learner in the process of learning (13). Researches done by Borgstede et al. show that learners are divided into 4 groups based on the type of goal. They include mastery, performance, approach, avoidance, and the kind of learners; orientation of the goal affects the motivation of learning and behaviors related to them (14). Graves, et al. (15) found that high academic performance is related to high mastery goal orientation and that mastery goals are positive predictors of deep processing, perseverance, and attempt. Anxiety is one of the negative emotions that plays a main role in everybody’s life. Test anxiety is one type of anxiety that is understood and accompanied by academic evaluation and is one of the most important negative aspects of motivation, with unpleasant effects on students' performance (16). Researchers identify test anxiety as a common and important educational phenomenon that has a close relationship with academic performance and achievement in students (17). Attribution theory is one of the theories that explains individuals' understanding of the causes of events based on psychology (18). Therefore, identifying and examining the locus of control is of high importance in explaining the method of individuals' performance, especially academic achievement and progress motivation. Researchers believe that with changing and modifying attribution styles in individuals, their motivation, and performance can change (19). Planning and task management are also taken from self-regulation. Self-regulation is a mental process in which the learner uses a range of strategies such as self-evaluation, self-control, setting a goal, managing the time, and organizing (20). Research literature has strongly supported learners' usage of self-regulation processes in academic achievement (21). In other words, learning self-regulation not only enhances students' learning but also provides them some opportunities to actively manage processes such as goal setting, self-control, self-evaluation, and self-motivation (22).

Researches indicate that trainers should not just attend to students' energy, but they should also pay more attention to channels that propel this energy towards meaningful learning goals or achievement. Considering the multidimensionality of motivation, a mixture of theories should be used to make the educational psychology models of motivation more applicable and comprehensible. Despite the importance that is mostly attributed to motivation in schools, only a few studies have examined multidimensional and general attitudes to cognitive and behavioral dimensions of motivation. Hence, testing multidimensional models in academic motivation and examining these factors can practically have a distinguishing effect compared to the previous researches in this field. The aim of the present study was to examine the effectiveness of multidimensional motivational interventions based on Martin's cyclic model on cognitive and behavioral dimensions of motivation in ninth grade students. 

## Materials and Methods

This was a quasi-experimental study with pretest, posttest, one-month follow-up, and control group. To examine the short-term effects, data of one-month interval were gathered. All female ninth grade students of the first shift in Rasht constituted the statistical population of the study. The sample included 90 individuals (45 individuals in the experimental group and 45 in the control group) who were included in the research based on the following criteria: being a ninth-grade student, willing to participate in the study, and not having severe family problems ( divorce)divorce, disease, addiction, or death of relatives). Exclusion criteria included absence of more than 3 sessions, dissatisfaction with continuing the participation in the rest sessions, and disease and other uncontrollable conditions. Available sampling method was used to select the participants. Firstly, 2 schools were selected, and then, all the ninth-grade students of these schools were asked to complete the demographic information form and declare their consent to attend group counseling sessions. Among students who were willing to participate in the sessions, those individuals who had similar socioeconomic condition were selected; moreover, it was decided that which school be considered as the experimental group and which school as the control group. To control the emission effect and prevent its effect on the results of the intervention, we selected the experimental and control groups were selected from 2 different schools. The intervention was done by the researcher The researcher conducted the intervention for fifteen 60-minute sessions and 3 times in a week, and it the sessions lasted nearly 2 months. Follow-up was done at the end of the third month. Of the experimental group, 10 participants and 2 from the control group were excluded from the study because of absence of more than 3 sessions and absence in the posttest and follow-up. Finally, the data related to 78 participants (35 participants in the experimental group and 43 in the control group) were analyzed.

Academic Self-Efficacy Beliefs Questionnaire: Zajacova et al., (19) have expanded the new version of Academic Self-efficacy Beliefs Questionnaire. In this questionnaire, the concept of academic self-efficacy beliefs were evaluated through 27 tasks related to the university or school. In this questionnaire, the participants were asked to identify the level of their certainty in doing each of university tasks successfully based on a 10-degree Likert scale. In a study by Shokri et al. (20), the internal consistency coefficient of the general factor of academic self-efficacy beliefs found to be 0.94. In the present study, the total score of academic self-efficacy was used. 

Achievement Goal Questionnaire (AGQ-R): Elliot and Murayama have expanded the revised version of Achievement Goal Questionnaire. The revised version has 12 items and 4 dimensions including tendency mastery-orientation goal, avoidant mastery-orientation goal, tendency performance goal, and avoidant performance goal (21). In a research by Hokmi & Shokri, the internal consistency coefficients were obtained 0.82, 0.80, 0.88 and 0.90, respectively for each dimension of goal mastery/tendency, mastery/avoidant, performance/tendency, and performance/avoidant. In the present study, just approach mastery goal and avoidant mastery goal were considered (22).

*Test Anxiety Inventory:* To identify the level of test anxiety, Sarason's Test Anxiety Inventory with 37 two-option items was used. The more the score of the individuals, the higher the level of anxiety in them. According to the study by Nezamdoost and Fereidooni, validity coefficient of this test was 0.76 based on Chronbach's alpha. In the present study, just the total score of test anxiety was used (23, 24).

Rutter's Intrinsic-Extrinsic Control Scale: This scale was made by Rutter in 1966. It has 29 items and each item has 2 sentences; each item is scored 0 or 1. The average of the reliability of this scale was reported to be 0.61 using Kuder Richardson and split-half methods and the reliability of retest with a 2- month interval was reported to be 0.7(25).

Learning Strategies Questionnaire (LSQ): This questionnaire was made by Weinstein and Mayer in 1986 and has 5 strategies, 77 items, and 10 scales. It was rebuilt by Vahedi in 5 components and after reviewing in 1997, some changes were made in the questionnaire and the components of the questionnaire changed into 6 components from 5 components. They included rehearsal, elaboration, organization, planning, supervision, control, and regulation that was added as a new component. In the present study just 2 dimensions of learning strategies and motivation were used (26).

***Implementation and Data Collection***

To examine the effect of multidimensional motivation interventions, an educational package of cognitive-behavioral dimensions based on Martin's theory (2008) was used. For each component, 3 sessions were considered, and considering the introductory meeting and pretest, posttest, and follow-up, there were 18 sessions.

In the present study, descriptive statistics (mean and standard deviation) and inferential statistics (repeated measures multivariate variance analysis) were used to analyze the data.

## Results

The findings revealed that the mean and standard deviation of the age of the participants in the experimental group were 15.31±0. 47, and 15.53±0.50 for participants in the control group. Moreover, the mean and standard deviation of the grade point average of participants were 17.35±2.03 in the experimental group and 17.14±1.90 in the control group. Table 2 presents the mean and standard deviation of the studied dimensions based on group and the test. To examine the effectiveness of multidimensional motivation interventions on cognitive and behavioral dimensions of motivation, repeated measures multivariate variance analysis was used. Therefore, first, the assumptions of this test were examined for each variable. The results of M-Box test for examining the assumption of homogeneity of covariance matrix showed that this assumption was not observed for studied components (M-Box = 645.269, F231,16159.33= 1.953, P<0.001). Considering the large sample size in the 2 groups, it can be stated that this test was persistent to not observing this assumption. Then, the assumption of sphericity was implemented for all variables using Muchley test, and the results showed that this assumption was observed for tendency mastery goal and cognitive strategies ( P>0.05) and was not observed for self-efficiency, avoidant mastery goal, test anxiety, source of control, and motivation (P<0.001).

Therefore, corrected results of Greenhouse Gaser were used. Then, the assumption of homogeneity of error variance was examined using Leven Test, and the results of this analysis showed that this assumption is observed for avoidant mastery goal, test anxiety, source of control, cognitive learning, and motivation in all 3 stages (P>0.05) and was not observed for self-efficacy and mastery goal (P<0.05).

**Table1:** Intervention modules and component summary based on Martin's Theory

**Sessions**	**The Content of the Sessions**
	Introduction, students getting to know each other, stating the rules of the sessions, and implementing pre-test
**The first to the third ** **sessions(self-efficacy)**	Challenging negative thoughts, identifying the ways of success at school, and individual's strengths and talents, and the way of applying them
**The fourth to the sixth ** **sessions (mastery goal)**	Examining the ways of achieving goals, active learning, changing the reasons for learning, and the way of applying them
**The seventh to the ninth ** **sessions**	Getting familiar with meditation techniques, preparing for the exam, and the way of applying them
**The tenth to the twelfth ** **sessions( the source of the ** **control)**	Identifying the reasons of previous academic results, identifying the capacity of controlling them, and focusing on controllable things and the way of applying them
**The thirteenth to the fifteenth ** **sessions( task management)**	Getting familiar with the appropriate conditions for studying, better use of time, expanding the time of studying during the week, and the way of applying them
	Implementing posttest
	Implementing follow-up( after 1 month)

**Table2:** The Mean and Standard Deviation of the Studied Dimensions Based on Group and Time of Implementing the Test

**Component**	**Control Group M(SD)**			**Experimental Group M(SD)**		
	Pretest	Posttest	Follow-up	Pretest	Posttest	Follow-up
Self- efficacy	185.97(41.66)	184.60(32.82)	185.04(36.7)	174.54(29.50)	204.20(17.49)**	190.74(23.10)
Approach mastery goal	13.88(1.41)	13.60(1.44)	13.06(1.93)	12.60(1.83)**	13.88(0.96)	13.08(1.56)
Avoidant mastery goal	11.39(2.18)	11.53(2. 06)	10.95(2.10)	10.22(2.28)*	12.57(1.44)*	11.08(1.80)
Test anxiety	55.48(5.63)	55.09(4.57)	55.34(5.52)	53.91(6.21)	45.51(4.03)**	49.25(5.08)**
Source of control	11.25(2.35)	11.02(2. 70)	11.09(2.29)	12.25(2.40)	9.85(2)*	10.80(2.63)
Cognitive strategies	41.55(4.30)	42(4.18)	40.93(4.02)	38.37(4.60)**	45.54(3.27)**	43.82(3.44)**
Motivation	77.55(8.16)	82.39(8.61)	77.97(8.36)	75.88(7.63)	87.28(4.78)**	78.34(7.03)

** : P<0/0001,

* : P< 0/05

**Table3:** The results of Wilks Lambda Tests to evaluate the effectiveness of intervention

		**Wilks lambda**	**F**	**P**	**Ƞ** ^2^
**Between-group**	Group	0.551	8.145	0.001	0.45
**Within-group**	Time	0.074	55.909	0.001	0.93
	Time & group	0.120	32.97	0.001	0.88

**Table4:** The Results of the ANOVA for evaluate differences in cognitive-behavior motivation components in both control and experimental groups

	**F**	**Df1**	**Df2**	**P**	**Ƞ** ^2^
Self- efficacy	79.253	1.47	111.73	0.001	0.510
Approach mastery goal	12.694	2	152	0.001	0.143
Avoidant mastery goal	39.167	175	133.17	0.001	0.340
Test anxiety	74.816	1.45	110.67	0.001	0.496
Source of control	18.708	11.85	140.78	0.001	0.189
Cognitive strategies	92.319	2	152	0.001	0.548
Motivation	126.352	1.86	141.75	0.001	0.624

**Figure1:**
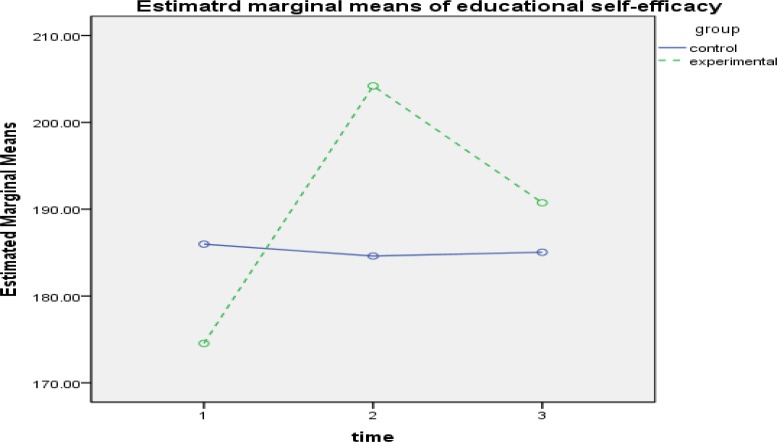
The Interactive Effect of Time-Group to evaluate the effectiveness of intervention in Self-Efficacy Component

**Figure2:**
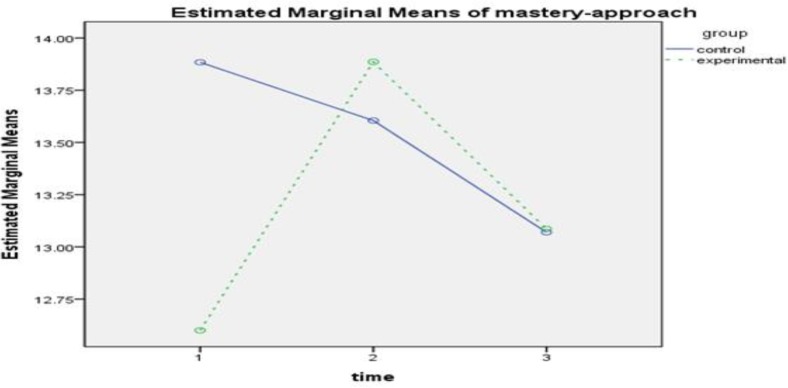
The Interactive Effect of Time-Group to evaluate the effectiveness of intervention in Approach Mastery Goal Component

**Figure3:**
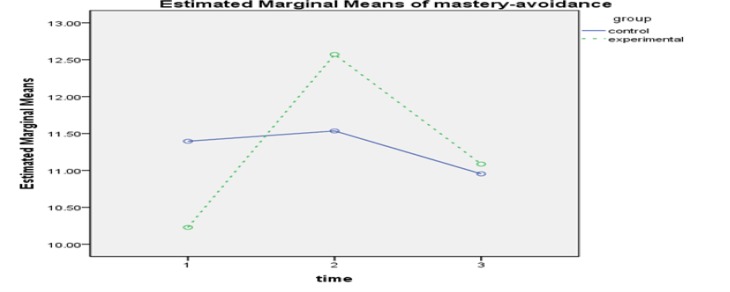
The Interactive Effect of Time-Group in Avoidant Mastery Goal Component

**Figure4:**
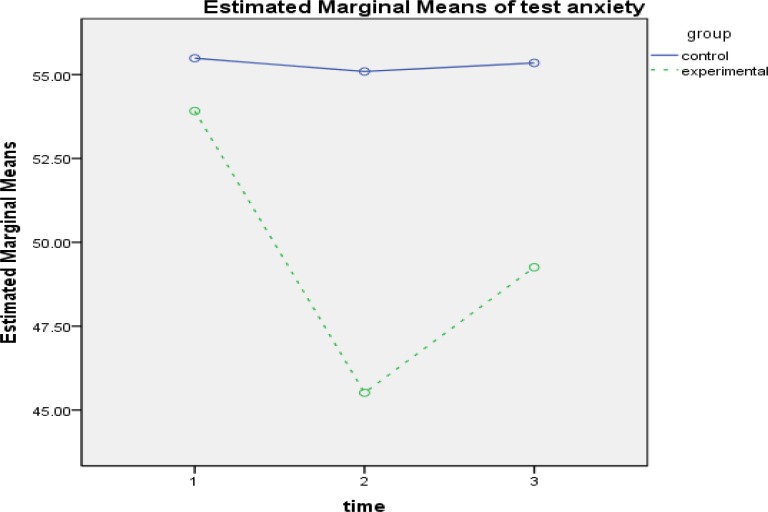
The Interactive Effect of Time-Group to evaluate the effectiveness of intervention in Test Anxiety Component

**Figure5:**
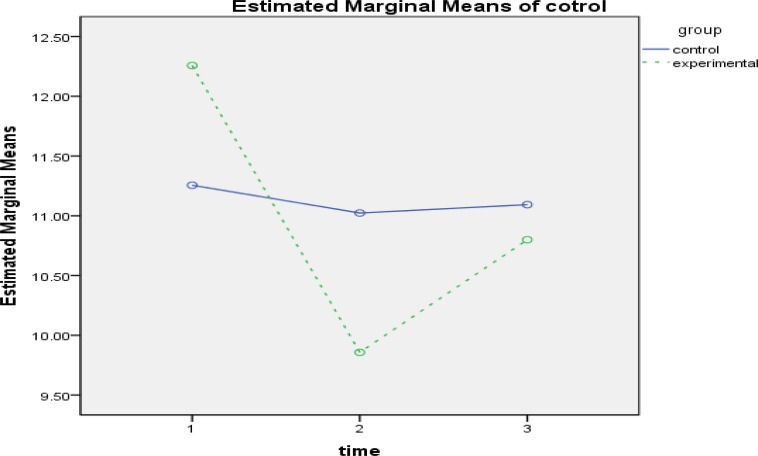
The Interactive Effect of Time-Group to evaluate the effectiveness of intervention in Locus of Control Component

The results of multivariable tests (Table 3) showed revealed that multidimensional interventions was were significant in between-group examination in the factor of group ( Wilks Lambda = 0.551,F = 8.145,P<0.001, ƞ2 = 0.45), and in within-group examinations in the factor of time (Wilks Lambda = 0.074, F = 55.909, P<0.001, ƞ2= 0.93), and in the interactive effect of time and group (Wilks Lambda = 0.120,F = 32.97,P<0.001, ƞ2 = 0.88).

between-group effect test did not show a significant difference between experimental and control groups in self-efficacy( F1,76=0.416, P = 0.521), tendency mastery goals (F1,76 = 1.066, P = 0.305), avoidant mastery goal (F1,76= 0.001, P = 0.999), source of control(F1,76= 0.09, P = 0.756), cognitive strategies (F1,76= 1.612, P = 0.208) and motivation (F1,76=0.543, P = 0.463), ). Moreover a significant difference was observed between experimental and control groups in test anxiety (F1,76 = 26.50, P = 0.0001).

The results of within-group effect test (Table 4) showed that the effect of time for self-efficacy(FGG1.47 ,111.73= 79.253,P<0.001, ƞ2 = 0.510), tendency mastery goal (FSA2,152=12.694,P<0.001, ƞ2 = 0.143), avoidant mastery goal(FGG1.75,133.17 = 39.167, P<0.001, ƞ2 = 0.340), test anxiety(FGG1.45,110.67= 74.816, P<0.001, ƞ2 =0.496), source of control(FGG1.85, 140.78=18.708, P<0.001, ƞ2 =0.198), cognitive strategies( FSA2,152= 92.319,P<0.001, ƞ2 = 0.548) and motivation( FGG1.86,141,75= 126.352,P<0.001) is was significant. The interactive effect of time and group was also examined, and the results of comparing the 2 groups in 3 stages of evaluation showed that in academic self-efficacy in pretest, there was not a significant difference between the groups. However, a significant difference was observed between the mean of the 2 groups in posttest, and this difference was in the interest of the experimental group (d = 19.59, P<0.01). The difference of the mean of the 2 groups was not significant at follow-up (Figure1). In tendency mastery goal, a significant difference was found just in pretest (d = -1.28, P<0.001). Although the amount of mastery goal has increased in participants of experimental group in posttest, it was not significantly different (Figure 2). In avoidant mastery goal, a significant difference was obtained between the 2 groups in pretest (d = -1.17, P<0.01) and posttest (d = 1.04, P<0.01), but this difference was not significant at follow-up (Figure 3). In test anxiety, no significant difference was found in pretest, but a significant difference was observed between the 2 groups in posttest (d = -9.58, P<0.001) and follow-up (d = -6.09, P<0.001), and this difference indicated reduction of test anxiety in the experimental group (Figure 4). In locus of control, no significant difference was observed between the 2 groups. There was a significant difference between the mean of the 2 groups in posttest, and this difference was in the interest of the experimental group (d = -1.17, P<0.01). However, the difference of the mean of the 2 groups was not significant at follow-up (Figure 5). In cognitive strategy, the difference of the 2 groups was significant in pretest (d = -3.18, P<0.001), posttest (d = 4.61, P<0.001), and follow-up (d = 2.89, P<0.001), and this difference indicated increasing this strategy in experimental group. In motivation, there was not a significant difference between the 2 groups in pretest. A significant difference was observed in the mean of the 2 groups in posttest, And this difference was in the interest of the experimental group (d=4.89, P<0.001), moreover, The mean of the 2 groups was not significant in follow-up. 

## Discussion

According to theoretical approach of Pintrich and Martin, to increase academic motivation in students, one factor that elaborates a set of cognitive and behavioral factors should be considered. In the present study, the effectiveness of multidimensional motivation intervention based on Martin's theoretical model was examined and the results revealed that providing 15 educational sessions in case of cognitive and behavioral components of motivation caused a significant difference in pretest and posttest of the experimental group compared to the control group. Moreover, even in some cases of the studied variables such as test anxiety and cognitive learning strategies, this difference was significant between posttest and follow-up, indicating the stability of the results of the intervention. However, about the components such as self-efficacy, mastery goal, source of control, and motivation, the difference between posttest and follow-up was not significant, indicating that for stability of these results, more time and accurate examinations are needed. 

Beliefs of self-efficacy are based on individuals' understanding of their personal performance, therefore, it is highly associated with individuals' performance (31). Green et al. (32) considered self-efficacy as an important factor in predicting academic performance in especial areas that can be developed using cognitive techniques. Hence, as it is expected, implementing multidimensional motivation interventions could increase academic self-efficacy in students of experimental group. Although cognitive techniques (challenging negative thoughts, identifying methods of academic success, and focusing on individuals' strengths) enhance this dimension in students, they seem to achieve stability in high self-efficacy. Thus, in addition to cognitive factors, environmental changes such as changes in students' homes and school environment should also be considered.

Martin, also, introduces mastery goals as one of the adaptive and effective cognitive dimensions on students' motivation and by providing multidimensional interventions of motivation, this component can be raised in students (4). In case of mastery goals, examining the data revealed a significant difference between experimental and control groups in pretest, and the mean of control group was higher than that of the experimental group. However, after providing multidimensional interventions, the means indicated an increase in tendency and avoidance mastery goals in the experimental group. Nonetheless, this difference was just significant in examining the posttest of avoidance mastery goal, and no difference was seen at follow-up. To explain these findings, it can be stated that in forming mastery goals, in addition to the role of students themselves, the educational system and feedbacks from family and teachers are also effective. Hence, multidimensional interventions cannot provide permanent effects on the types of students' goals, and to create this change, the support of other factors is necessary.

According to Martin's theoretical model, anxiety is an incompatible cognitive dimension that has a negative effect on students' motivation. Moreover, using cognitive therapeutic techniques, training relaxing, and getting familiar with the strategies of the test and taking the test, anxiety can be reduced in students, and therefore, motivation will increase in them (4). Students with test anxiety, often spend more time on studying compared to their classmates, but are not sure of their abilities and often fail to achieve favorable results. This issue generally decreases students’ motivation for more attempts and even can have negative effects on their motivation (33). The results of the present study have also shown that multidimensional motivation interventions, according to Martin's theoretical approach, resulted in reduction of anxiety in students of the experimental group, and this reduction was significant in posttest and follow-up, indicating the stability of the effect of intervention and the expanded role of the students themselves in controlling anxiety.

Attributed beliefs refer to how individuals justify their academic successes and failures. Studies have found that the locus of control in students and their performance expectations for success and failure can have a close relationship with motivation (34). The results of the present study also showed that multidimensional motivation interventions have increased the amount of locus of control in the experimental group between pretest and posttest, but this comparison was not significant between posttests and follow-up. Thus, it can be inferred from the results that multidimensional motivation interventions can increase the feeling of locus of control in students in a short time. However, as the locus of control is formed by our experiences, environmental, cultural, and social conditions and family structure, making fundamental changes need more time and supportive factors. Therefore, attribution-retraining techniques should be designed to have higher permanency and resistance to environmental changes.

In Marti's theory, also the skill of task management is a behavioral variable in which the individuals control their time, prioritize their homework, and provide appropriate conditions for their homework (4). In a study on high school students done by Labuhn et al., (35), it was found that those individuals, who had high self-regulation skills, had higher academic motivation and success. The results of the present study showed that providing multidimensional motivation interventions increases task management skills and learning and motivation strategies in students of the experimental group. However, the results revealed no significant differences between the posttest of the 2 groups in the component of motivation at the follow-up. The time of implementing the follow-up that was after New Year holidays could be mentioned and students might have needed less time to use the learnt techniques in managing the tasks.

## Limitations

The lack of a comprehensive questionnaire on motivation and sample size should be considered when generalizing the results of this study.

## Conclusion

For years, researchers of educational psychology and other authorities in this field have been concerned about lack of motivation and reduction of academic performance; and turning to multidimensional approaches is one of the achievements of them. Considering the theoretical and practical evidences and previous studies, it was found that cognitive and behavioral dimensions was associated with a vast range of motivation and academic variables, and providing training to students on these dimensions can positively affect students' motivation and academic performance. In addition, the follow-up results revealed that although providing training to students on cognitive and behavioral dimensions of motivation leads to a significant change in these dimensions, for stability of this effect, the family and school personnel should be trained to gain more sustained results. Therefore, considering the achieved results, it is recommended that authorities of education and educational consultants, based on the existing research evidence, use multidimensional approaches to boost motivation and increase students' academic performance. Moreover, authorities should increase the awareness of parents, teachers, and other educational authorities through holding in-service training courses.

## References

[1] Green J, Arief G, Martin A, Colmar S, Marsh H, MC Inerney D (2012). Academic motivation, Self-concept, Engagement, and performance in high school: Key processes from a longitudinal perspective. J of Adolesc.

[2] Ferda-Bedel E (2016). Exploring academic Motivation, academic self-efficacy and attitudes toward teaching in pre-service early childhood education teachers. j of Educ and train stud.

[3] Pintrich PR (2003). A motivational science perspective on the role of student motivation in learning and teaching contexts. J of Educ Psychol.

[4] Martin A (2008). Enhancing student motivation and engagement: The effects of a multidimensional intervention. Contemp Educ psychol.

[5] Akhlaghi M (2014). [The effectiveness of interventions in the multidimensional motivational - cognitive task value, mastery goal orientation and academic self-efficacy (In Persian)]. Master's thesis.

[6] Pooragha F, Talepasand S (2014). [Constructing and Validating the Multifactor Questionnaire of Academic Motivation in Students of Semnan – Specialized for the First Grade High School Students (In Persian)]. J of educ Meas and Eval Studi.

[7] Zamani M (2014). [The effectiveness of multidimensional motivational interventions - behavioral treatment progress and academic motivation (In Persian)]. Master's thesis.

[8] Honicke T, Broadbent J (2016). The influence of academic self-efficacy on academic performance: A systematic review. Educ Res Rev.

[9] Jaengaksorn N, Ruengtrakul A, Piromsombat C (2015). Developing self –efficacy and motivation to be a teacher scale Thai version. soc and behav sci.

[10] Denzine G, brown R (2015). Motivation to learn and Achievement.

[11] Bandura A, P M Van Lange, A W Kruglanski, E Higgins (2012). Social cognitive theory. Handbook of theories of soci psychol.

[12] Anderman EM, Patrick H, S. L. Christenson, A. L. Reschly C. Wylie (2012). Achievement goal theory, conceptualization of ability/ intelligence, and classroom climate.

[13] Li j Shien Ch (2016). A study on the Effects of multiple goal orientation on learning motivation and learning behaviors. Eurasia J of maths, Sci and Tec Educ.

[14] Borgstede Cv, Andersson M, Johnsson F (2013). Public attitudes to climate change and carbon mitigation—Implications for energy-associated behaviors. Energ Pol.

[15] Graves L M, Ruderman MN, Ohlott P J, Weber TJ (2012). Driven to work and enjoyment of work effects on managers’ outcomes. J of Manag.

[16] Kilmen S (2015). Why do the tests Make us Anxious?. Int j soc sci and educ.

[17] Mishra S, Chincholikar Kl (2014). A study of relationship of academic achievement with aptitude, attitude and anxiety. Int J of English lang, lit and Humanit.

[18] Kizgin Y, Dalgin T (2012). Attribution theory: attribution differences of students between success and failure circumstance, zonguldak karaelmas university. j socl sci.

[19] Haynes S T L, Clifton R A, Daniels L M, Perry R P, Chiperfield J G, Ruthing J C (2010). Attribution retraining: Reducing the likelihood of failure. Soc Psychol of Educ: An Int J.

[20] Gibbs R, Poskitt J Student Engagement in the Middle Years of Schooling (Years 7-10): A Lit Rev 2010; 02/08/2015.

[21] Williamson G (2015). Self-regulated learning: an overview of metacognition, motivation and behavior. j of Initial Inquiry.

[22] Fang N (2014). Correlation between students motivated strategies for learning and academic achievement in an engineering dynamics course. Global j of Eng educ.

[23] Zajacova A, Lynch S M, Espenshade TJ (2005). Self-efficacy, Stress, and Academic Success in college. Res in Hig Educ.

[24] Shekari A, Tamizi N, Abdollahpoor M, Taghvai nia A (2015). [The reliability and validity of the revised version of the questionnaire aims at students' progress (in Persian)]. J cog strat in learn.

[25] Elliot A J, Murayama K (2008). On the measurement of achievement goals: Critique, illustration, and application. J of Educ Psychol.

[26] Hokmi S, shekari A (2015). [The relationship between achievement goal orientation and academic well-being: emotions mediate the effects of progress (in Persian)]. J of educ Meas and Eval Studi.

[27] Feridoni M (2005). [Examine the feasibility, reliability and validity of the norm finding Sarason Test Anxiety Test (in Persian)]. Master's thesis.

[28] Nezamdoost A (2004). [Feasibility study to assess the validity of narrative and soft FINDING Sarason test anxiety (in Persian)]. Master's thesis.

[29] Shahande M (2008). [Personality assessment tests and questionnaires (in Persian)].

[30] Khadivzade t, seif A, Valaee N (2002). [Study strategies and study skills and academic achievement of students of Mashhad University of Medical Sciences (in Persian)]. Researcher, martyr Beheshti University of Medical Sciences.

[31] Kurt T, Duyar I, Calik T (2011). Are we legitimate yet? A closer look at the casual relationship mechanisms among principal leadership, teacher self-efficacy and collective efficacy. J of Manag Dev.

[32] Green J, Arief G, Martin A, Colmar S, Marsh H, MC Inerney D (2012). Academic motivation, Self-concept, Engagement, and performance in high school: Key processes from a longitudinal perspective. J of Adolesc.

[33] Soffer EM (2008). [Elementary Students ' Test Anxiety in Relation to the Florida Comprehensive Assessment Test (FCAT) ]. A Thesis submitted to the Department of Family and Child Sciences in partial fulfillment of the requirements for the degree of Masters in Science.

[34] Nuttal J (2016). Relationship between motivation attribution and performance expectancy in children's reading. the Plymouth student scientist.

[35] Labuhn A S, Zimmerman B J, Hasselhorn M (2010). Enhancing students’ self-regulation and mathematics performance: The influence of feedback and self-evaluative standards. Metacog and Learn.

